# SPA inhibits hBMSC osteogenic differentiation and M1 macrophage polarization by suppressing SETD2 in acute suppurative osteomyelitis

**DOI:** 10.1038/s41598-024-63219-0

**Published:** 2024-06-03

**Authors:** Dongsheng Zhu, Feng Chen, Hongjia Qiang, Han Qi

**Affiliations:** 1 Department of Pediatric Surgery, The First People’s Hospital of Lianyungang, Lianyungang, Jiangsu Province China; 2 Department of Pediatric, Luodian Hospital, Shanghai, China; 3 Department of Emergency Surgery, The Second People’s Hospital of , Lianyungang, Jiangsu Province China

**Keywords:** SETD2, M1 macrophage polarization, Osteomyelitis, Osteogenic differentiation, Molecular biology, Medical research

## Abstract

To clarify the impact of SETD2 on macrophage function in pediatric patients with acute suppurative osteomyelitis and to elucidate the precise underlying mechanism. To gain insights into the potential functions of SETD2, a comprehensive study was conducted utilizing a co-culture model of human bone mesenchymal stem cells (hBMSCs) and bone marrow-derived macrophages (THP-1). A range of techniques were employed, including quantitative polymerase chain reaction, western blotting, ELISA, alkaline phosphatase activity assays, alizarin red S staining, luciferase reporter gene assays, and chromatin immunoprecipitation, to unravel the intricate interactions and molecular mechanisms involving SETD2 in this system. It was observed that SETD2 expression was reduced in THP-1 cells stimulated by staphylococcal protein A (SPA). Furthermore, the downregulation of SETD2 resulted in elevated M1 macrophage polarization and glycolysis, effects that were mitigated by SPA stimulation. Notably, SPA-stimulated THP-1 cells exhibited an increase in HIF-1α expression, which exhibited an inverse correlation with SETD2 levels. Moreover, it was discovered that SETD2 functioned as a catalyst for H3K36me3 and bound to the HIF-1α gene, which, in turn, regulated HIF-1α expression. Furthermore, the suppression of HIF-1α abrogated the consequences of SETD2 downregulation on glycolysis and M1 macrophage polarization. Lastly, the study demonstrated that M1 macrophage polarization serves as a mediator for BMP4’s inhibitory effect on osteogenic differentiation of hBMSCs. This research has uncovered a previously unknown role of SETD2 in macrophages during osteomyelitis, revealing its significance in the pathogenesis of this condition. These findings suggest SETD2 as a novel target for the treatment of osteomyelitis.

## Introduction

Osteomyelitis, an inflammation of the bone resulting from an acute bacterial infection, particularly affects children, with an incidence rate of 0.2–1.3%^1^. Osteomyelitis is a major global health burden since it is regarded as a prevalent condition with substantial healthcare-related costs and the primary cause of hospital death^2^. Despite the challenges in diagnosing acute osteomyelitis at an early stage, prompt treatment is crucial to prevent bone loss and mitigate long-term complications. Septicemia, bone deterioration, or persistent osteomyelitis can result from failing to identify and treat such infections^3^. Despite progress in osteomyelitis treatment, persistent issues such as difficulties with weight bearing, limited range of motion in affected extremities, limb length disparities, and pathologic fractures still remain as potential complications^4^. Therefore, research into osteomyelitis treatment approaches is essential.

The majority of patients with osteomyelitis are able to endure the initial inflammatory response phase and transition into the prolonged immunosuppressive phase due to advancements in medical technology^5^. Macrophages, crucial innate immune cells, play a pivotal role by eliminating and phagocytizing microorganisms in the pathogenesis of osteomyelitis^6^. According to reports, controlling macrophage polarization and function improves osteomyelitis development and prognosis^7^. Moreover, it has been shown that during inflammation, macrophages’ metabolic pattern changed from oxidative phosphorylation to glycolysis^8^. Hypoxia-inducible factor 1α (HIF-1α) emerged as a pivotal factor throughout the inflammation process, highlighting its significance in the pathogenesis of this condition^9^. In sepsis, HIF-1α was identified as a key activator in macrophages, promoting the expression of glycolysis-related proteins and thereby facilitating the transition towards glycolysis^10^. Currently, it remains unclear whether HIF-1α is activated during osteomyelitis.

SET domain-containing 2 (SETD2) is a methyltransferase responsible for trimethylation of histone H3 at lysine 36 (H3K36me3) and regulates diverse biological processes by modulating the methylation status of H3K36^11^. Despite its importance, the role of SETD2 in osteomyelitis remains enigmatic. However, recent investigations have shed light on the fact that methylation modification plays a crucial role in regulating the stability of HIF-1α^12^. Furthermore, a previous study suggested at the possibility that SETD2 could serve as a novel metabolic regulator^13^. Based on these findings, we hypothesized that SETD2 might regulate the expression of HIF-1α, thereby modulating macrophage glycolysis in osteomyelitis. This regulation could potentially impact macrophage function and polarization, thus influencing the pathogenesis of osteomyelitis.

## Materials and methods

### Cell culture

The macrophage cell line THP-1, derived from human monocytes, was obtained from the prestigious American type culture collection (TIB-202, ATCC, USA). Human bone mesenchymal stem cells (hBMSCs) were sourced from Ningbo Mingzhou Biotechnology Co., LTD (MZ-2720, Ningbo, China). Both the two cell lines were cultured in RPMI 1640 medium (Corning, NY, USA) that was supplemented with 10% fetal bovine serum (FBS; Gibco BRL, Gaithersburg, MD, USA) to provide optimal growth conditions. The cells were maintained in a humidified incubator, set at 37 °C with 5% CO_2_. THP-1 cells were then exposed to different concentrations of staphylococcal protein A (SPA; Aladdin Corporation, Shanghai, China) at 0, 0.5, 1, or 2 μg/ml for a duration of 48 h. To assess the osteogenic potential of hBMSCs in a macrophage-mimicking microenvironment, the cells were seeded with THP-1 macrophages in a specialized transwell chamber featuring a fine mesh of 0.8 μm pores, ensuring a controlled yet interactive co-culture ratio of 1:10. THP-1 cells were incubated in the upper chamber of the transwell system, while hBMSCs were cultured at the bottom chamber. Both chambers were maintained for duration of 1 week, during which the medium was replaced twice weekly. To delve deeper into the polarization phenotype of macrophages, the culturing conditions were reversed; this time, hBMSCs were placed in the upper chamber, while macrophages were seeded in the lower chamber. The quantification of M1-like biomarkers, such as iNOS, IL-6, IL-1β, and TNF-α, was achieved through either quantitative polymerase chain reaction (qPCR) or enzyme-linked immunosorbent assay (ELISA), providing precise insights into their expression levels.

### RNA extraction and qPCR

Utilizing the TRIzol reagent from Thermo Fisher Scientific (Invitrogen, California, USA), RNA was isolated from both hBMSCs and THP-1 cells, ensuring high-quality RNA extraction for subsequent analyses. After RNA isolation, the PrimeScript RT reagent kit from Thermo Fisher Scientific, Inc. was used to convert the RNA into complementary DNA. Subsequently, qPCR was performed on the Applied Biosystems Prism 7900 system, using the SYBR-Green master mix kit from the same manufacturer, Thermo Fisher Scientific, Inc. The entire process was carried out according to the manufacturer’s instructions. A comprehensive list of primers employed in this study is provided in Table 1.

**Table 1:** Reverse transcription quantitative polymerase chain reaction primers.

Gene	Primer sequence(5ʹ–3ʹ)
HIF-1α (human)	F: CATAAAGTCTGCAACATGGAAGGT R: ATTTGATGGGTGAGGAATGGGTT
HIF-1α (mouse)	F: TGATGTGGGTGCTGGTGTC R: TTGTGTTGGGGCAGTACTG
SETD2 (human)	F: TGCTTCTAGTCGATTTTTGCCC R: AGGGTTTGGAGTATCACTTTGC
SETD2 (mouse)	F: ATA ATA GGG AGC CGA CAG R: AAT CAG GAA GGG CAC TAC
iNOS (human)	F: TCTTGGTCAAAGCTGTGCTC R: CATTGCCAAACGTACTGGTC
iNOS (mouse)	F: TTG CCA CGG ACG AGA CGG ATAG R: GGG CAC ATG CAA GGA AGG GAAC
OSX (human)	F: GAAGAAGCTCACTATGGCTC R:GAAAAGCCAGTTGCAGACGA
OSX (mouse)	F: CACCCTTCCCTCACTCATTT R:CCTTGTACCACGAGCCATAG
RUNX2 (human)	F: TGGTTACTGTCATGGCGGGTA R:TCTCAGATCGTTGAACCTTGCTA
RUNX2 (mouse)	F: CGACAGTCCCAACTTCCTGT R:CGGTAACCACAGTCCCATCT
GAPDH (human)	F: CATCACTGCCACCCAGAAGACTG R:ATGCCAGTGAGCTTCCCGTTCAG
GAPDH (mouse)	F: CATCTCCTCCCGTTCTGCC R:GTGGTG-CAGGATGCATTGC

### Western blotting

Western blotting was executed according to our previously published protocol, ensuring consistency and reproducibility in the analysis^14^. The Pierce^™^ ECL western blotting substrate, catalog number 32109, obtained from Thermo Fisher Scientific, Inc., was utilized to reveal the antibodies bound to the protein. The assessment of protein levels was performed using a chemiluminescence detection system (GE Healthcare Biosciences, USA). A comprehensive list of antibodies utilized in this study is provided in Table 2.

**Table 2:** The list of the antibodies used for western blotting.

Antibody	Catalog number	Host	Dilution ratio	Company
SETD2	ab239350	Rabbit	1:1000	Abcam
β-actin	ab8227	Rabbit	1:5000	Abcam
HK2	ab209847	Rabbit	1:1000	Abcam
HIF-1α	ab179483	Rabbit	1:1000	Abcam
H3K36me3	ab282596	Rabbit	1:1000	Abcam
Histone H3	ab308373	Rabbit	1:1000	Abcam
anti-rabbit lgG	ab150077	Goat	1:4000	Abcam

### Cell transfection

Custom-designed siRNA oligonucleotides for SETD2 (si-SETD2) and HIF-1α (si-HIF-1α) as well as over-expression oligonucleotides for SETD2 (oe-SETD2) were purchased from GenePharma Inc (Shanghai, China). For knockdown or over-expression experiments, these oligonucleotides were efficiently transfected into THP-1 cells using lipofectamine 3000 (Thermo Fisher Scientific, USA).

### Measurement of extracellular acidification rate (ECAR)

The ECAR was measured using the Seahorse XF Glycolysis Stress Test Kit (Agilent Technologies, USA), along with the Seahorse Extracellular Flux Analyzer XF96 (Seahorse Bioscience, USA). The entire procedure was carried out according to the manufacturer’s guidelines to ensure accurate results. Cells were initially seeded onto 96-well plates and incubated overnight at 37 °C to ensure proper cell attachment and growth. Subsequently, the cells were harvested for the measurement of extracellular acidification rate (ECAR). To assess ECAR, the baseline was recorded, followed by the sequential addition of glucose (1 μM), oligomycin (1 μM), and 2-deoxyglucose (0.5 μM) to each well. This approach allowed for a comprehensive evaluation of ECAR in response to these metabolic modulators.

### ELISA

To quantify interleukin (IL)-6, tumor necrosis factor (TNF)-α, IL-1β, bone morphogenetic protein (BMP)4, and C-reactive protein (CRP) levels in the medium or serum, we employed ELISA kits specifically designed for each protein (R&D, Minneapolis, USA). The procedures were followed strictly according to the manufacturer’s guidelines to ensure accurate and reproducible results.

### Luciferase reporter assays

The HIF-1α promoter fragment originating from *Homo sapiens* was amplified and subsequently cloned into the pGL3-basic vectors for further analysis. The THP-1 cells were transfected with either the empty vector or plasmids expressing SETD2, along with the pGL3-HIF-1α promoter reporter vector, utilizing the lipofectamine 3000 transfection reagent to ensure efficient gene delivery. Following a 48 h incubation period, the luciferase activity was assayed using the Dual-Luciferase Reporter Assay System (GenePharma, China), adhering strictly to the manufacturer’s guidelines for accurate quantification.

### Chromatin immunoprecipitation (ChIP) assays

ChIP assays were carried out using the Magnetic ChIP Kit (Millipore, USA), following the manufacturer’s instructions for optimal results. Prior to ChIP assays, THP-1 cells were immobilized with 1% formaldehyde and subsequently fragmented using micrococcal nuclease (MNase) and sonication. For immunoprecipitation, antibodies specific for H3K36me3 (MA5-24687, ThermoFisher Scientific, USA) or a negative control IgG (30000-0-AP, Proteintech, USA) were employed. Following washing and reverse cross-linking steps, the precipitated DNA was amplified using specific primers and quantified via PCR. To ensure accurate comparisons, all obtained values were normalized relative to the input DNA.

### Alizarin red-sulfate (ARS) staining assay

To assess mineralization, the ARS staining method was employed. Initially, the cells were fixed with 4% paraformaldehyde for 30 min, followed by two washes with phosphate buffered saline (PBS) to remove any residual fixative. After fixation and washing, the cells were incubated with the ARS stain solution (Leagene Biotech., China) for 10 min at room temperature. Subsequently, 10% acetic acid (Sigma, USA) was employed to elute the ARS. The optical density (OD) was measured at an absorbance of 540 nm for both quantitative assessment and imaging purposes.

### Alkaline phosphatase (ALP) enzyme assay

After successfully inducing osteogenesis, we gathered 1 million hBMSCs and lysed them using PIPA lysis buffer (Beyotime Biotechnology, China) for a duration of 5 min. After lysing the cells, a centrifugation step was carried out at 4 °C for 3 min to separate the supernatant. Following this, 5 mL of ALP substrate buffer (BD Biosciences Clontech, USA) was added to the supernatant for further enzymatic analysis. The activity of ALP was assayed through a colorimetric analysis of the reaction mixture, which was incubated for 1 h to allow for sufficient enzymatic reaction.

### SETD2 inhibitor EZM0414

The stock solution of EZM0414 (HY-144858, MedChemExpress LLC, Shanghai, China) was prepared by dissolving it in dimethyl sulfoxide (DMSO) to a concentration of 10 mM. This stock solution was then stored at − 20 °C for future use. The mice were orally dosed with EZM0414 at a concentration of 50 mg/kg/day, strictly adhering to previously validated dosing guidelines to maintain consistency and accuracy^15^.

### Mouse osteomyelitis model

To induce osteomyelitis as a model for studying the disease, the mice utilized in this study were infected with *Staphylococcus aureus*, adhering strictly to previously validated procedures for infection induction^16^.

### Patients and specimens

Blood samples were obtained from children diagnosed with bacterial osteomyelitis. Positive bacteremia findings in osteomyelitis patients were further verified using at least one of the following methods: percutaneous puncture, surgical sampling, or blood culture. Additionally, blood samples were gathered from 12 children with bacterial osteomyelitis prior to any surgical intervention or treatment. Concurrently, blood samples were also collected from 12 healthy volunteers.

### Statistical analysis

We analyzed the obtained data using GraphPad Prism (version 8; GraphPad Software, USA), to extract meaningful insights and comparisons. To compare continuous variables between two groups, we utilized the Mann–Whitney U test. For comparing continuous variables among multiple groups, we employed the one-way ANOVA. Subsequently, to determine significant differences among the groups, we conducted the Dunnett’s post hoc test following the ANOVA analysis. P < 0.05 was considered statistically significant. All experiments were replicated three times to guarantee reproducibility and consistency of results.

### Ethical approval

The present study was granted approval by the Ethics Committee of the First People’s Hospital of Lianyungang.

## Results

### SETD2 effectively blocks the promotion of M1 macrophage polarization and glycolysis triggered by SPA in TPH-1 cells

**Figure 1:**
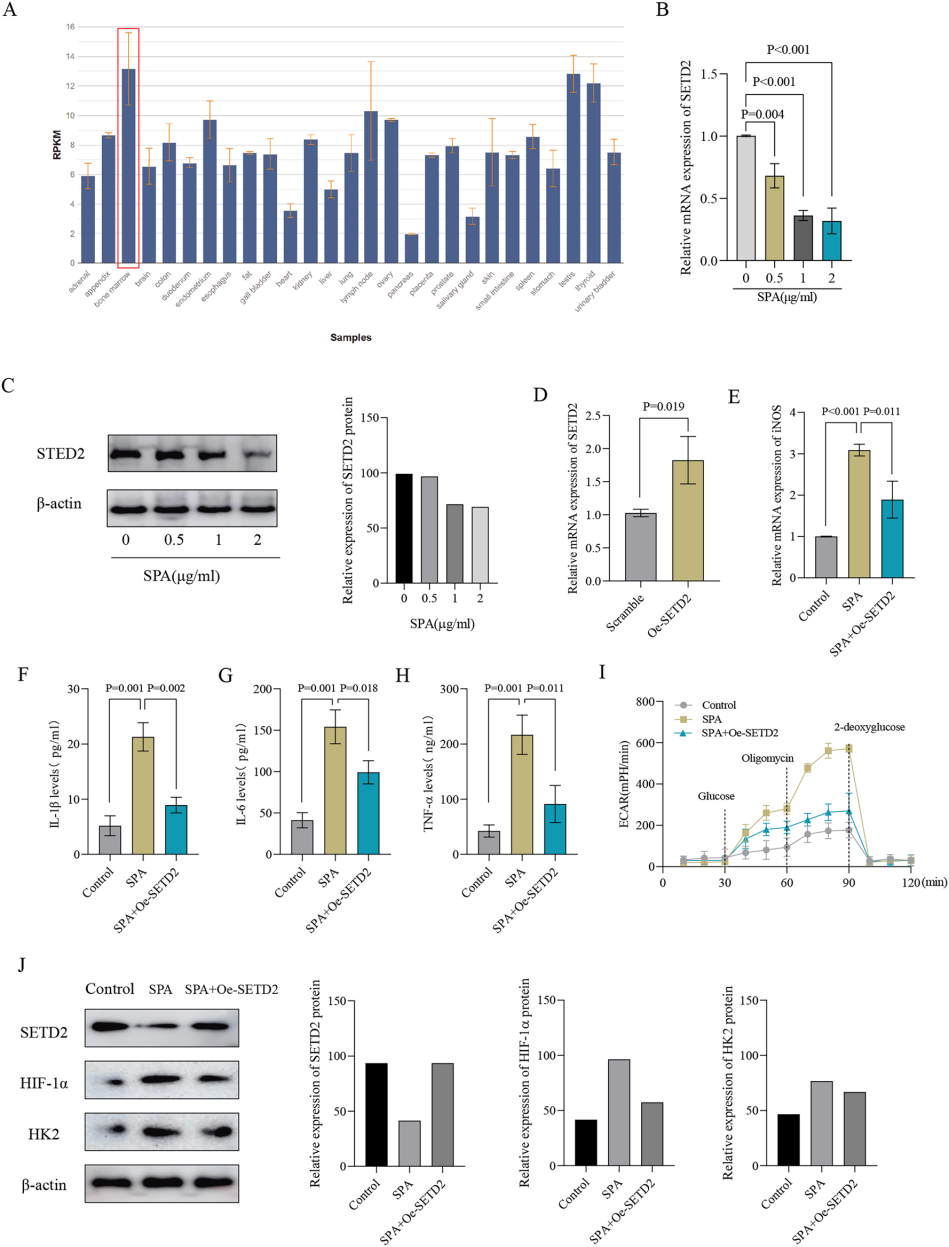
The overexpression of SETD2 suppresses SPA-induced M1 macrophage polarization and glycolysis in THP-1 cells. (**A**) Expression of SETD2 in various tissues of humans is shown in the NCBI database. (**B**) THP-1 cells were stimulated with SPA at varying concentrations (0, 0.5, 1, and 2 μg/ml) for duration of 48 h. Subsequently, the levels of SETD2 mRNA were quantified using qPCR. (**C**) THP-1 cells were exposed to varying doses of SPA (0, 0.5, 1, and 2 μg/ml) for 48 h. Following this, the levels of SETD2 protein were assessed through western blot analysis. (**D**) Overexpression of SETD2 in THP-1 cells. (**E**) The iNOS mRNA levels were determined by qPCR in THP-1 cells treated with either a SETD2 expression vector or an empty vector, in the presence of 1 μg/ml SPA for 48 h. Untreated cells served as the control for this experiment. (**F**–**H**) The levels of cytokines such as IL-1β, IL-6, and TNF-α in the cell supernatants were measured using ELISA, under the previously described conditions. (**I**) ECAR levels under the same conditions described above. (**J**) Illustrative western blot images depicting the expression patterns of SETD2, HIF-1α, and HK2 are presented, following the previously described experimental conditions.

We initially investigated the expression of SETD2 in various human tissues through the NCBI database and observed that SETD2 expression was particularly high in bone marrow (Fig. 1A). In our subsequent analysis, we evaluated the expression of SETD2 in TPH-1 cells treated with SPA. We noticed a considerable decrease in the expression of SETD2 at both the mRNA and protein levels in cells exposed to SPA at dosages ranging from 0 to 2 μg/ml (Fig. 1B, C). Therefore, 1 μg/ml SPA was selected for subsequent experiments. This concentration is consistent with previous literature reports for the construction of osteomyelitis cell models, ensuring comparability and reproducibility of the findings^17^. We overexpressed SETD2 in TPH-1 cells to assess its effect on cell function and phenotype. The overexpression of SETD2 was confirmed through qPCR analyses (Fig. 1D).Following a 48 h treatment with 1 μg/ml SPA, we observed an upregulation of M1 polarization markers iNOS, IL-1β, IL-6, and TNF-α in TPH-1 cells, indicating a shift towards a proinflammatory phenotype. Interestingly, the overexpression of SETD2 significantly suppressed the SPA-induced upregulation of M1 polarization markers iNOS, IL-1β, IL-6, and TNF-α in TPH-1 cells, suggesting that SETD2 plays a crucial regulatory role in the polarization of these cells (Fig. 1E–H). Additionally, SPA treatment led to an increase in ECAR, a marker of glycolytic activity. However, the overexpression of SETD2 inhibited this SPA-induced increase in ECAR (Fig. 1I). This suggests that SETD2 may regulate glycolytic metabolism in TPH-1 cells. We confirmed that overexpression of SETD2 inhibited the SPA-induced increase in the protein levels of HIF-1α and HK2 (Fig. 1J). HIF-1α serves as a crucial transcription factor regulating hypoxic responses, whereas HK2 functions as a key enzyme in the glycolysis pathway^12^. The present findings demonstrate that SETD2 potently blocks the SPA-induced promotion of M1 macrophage polarization and glycolysis in TPH-1 cells, indicating its regulatory role in cellular metabolism and inflammatory responses.

### Regulation of HIF-1α expression by SETD2-catalyzed H3K36me3

**Figure 2:**
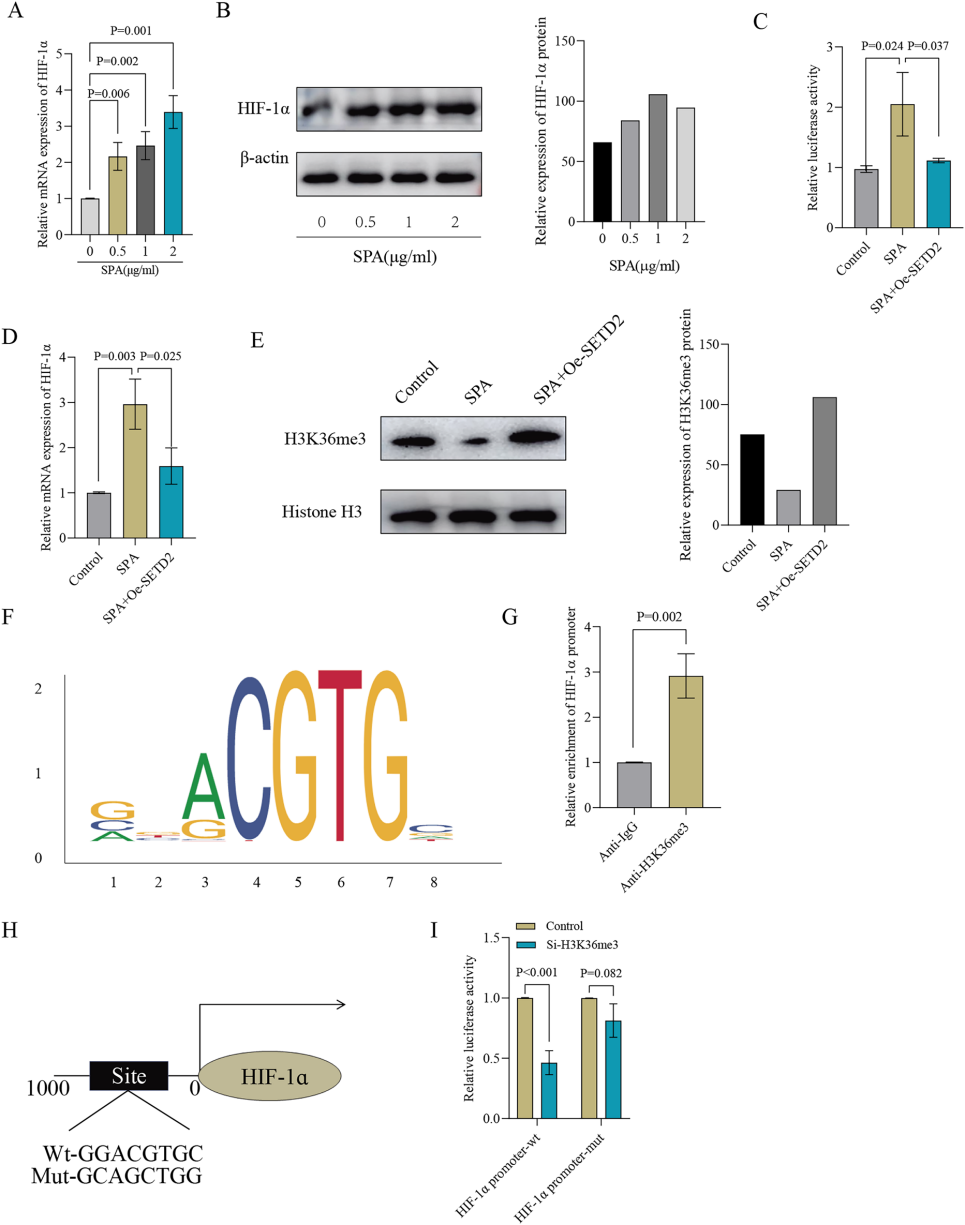
Regulation of HIF-1α expression by SETD2-catalyzed H3K36me3. (**A**) THP-1 cells were treated with varying concentrations of SPA (0, 0.5, 1, and 2 μg/ml) for 48 h, followed by quantification of HIF-1α mRNA levels using qPCR. (**B**) THP-1 cells were exposed to varying doses of SPA (0, 0.5, 1, and 2 μg/ml) for 48 h, and subsequently, the protein levels of HIF-1α were assessed by western blot analysis. (**C**) The luciferase activity was measured in THP-1 cells treated with either a SETD2 expression vector or an empty vector, in the presence of 1 μg/ml SPA for 48 h. Untreated cells were used as the control for this experiment. (**D**) The levels of HIF-1α mRNA were quantified by qPCR under the previously mentioned conditions. (**E**) The protein levels of H3K36me3 were assessed by western blot analysis, following the previously described conditions. (**F**) The binding motif for H3K36me3 was retrieved from the JASPAR database. (**G**) The affinity of H3K36me3 for the promoter region of HIF-1α was evaluated through a ChIP assay. (**H**) Putative binding locations for H3K36me3 in the transcriptional start site region of the HIF-1α gene. (**I**) A luciferase reporter assay was conducted to demonstrate the influence of H3K36me3 on the transcriptional activity of the HIF-1α gene.

To gain deeper insights into the mechanism underlying SETD2’s repression of HIF-1α expression, we examined the expression levels of HIF-1α in TPH-1 cells treated with SPA. Our findings revealed a dose-dependent upregulation of HIF-1α expression in TPH-1 cells upon SPA stimulation (Fig. 2A, B). To investigate the association between SETD2 and HIF-1α, a luciferase reporter assay was performed to assess the transcriptional activity of the HIF-1α promoter. The results obtained indicate that SPA triggers a significant enhancement in the transcriptional activity of the HIF-1α promoter, thereby suggesting that the regulation of HIF-1α expression by SPA occurs primarily at the transcriptional level. Moreover, the overexpression of SETD2 was found to attenuate the SPA-induced transcriptional activation of the HIF-1α promoter, indicating that SETD2 may exert its inhibitory effect on HIF-1α expression by suppressing its transcriptional activity (Fig. 2C). Additionally, our findings revealed that the overexpression of SETD2 effectively blocked the SPA-induced elevation in HIF-1α mRNA levels (Fig. 2D). This observation reinforces the conclusion that SETD2 regulates HIF-1α expression at the transcriptional level. The precise mechanism underlying SETD2’s repressive effect on HIF-1α expression remains to be fully elucidated and warrants further investigation. Apart from its observed impact on HIF-1α expression, SPA treatment was also found to decrease the levels of H3K36me3, a histone modification that is associated with transcriptional silencing. This reduction was reversed by overexpression of SETD2, indicating that SETD2 may regulate gene expression through histone modification (Fig. 2E). The binding motif of SETD2 was anticipated by utilizing the JASPAR database (Fig. 2F). The ChIP assay demonstrated a significant affinity between H3K36me3 and the HIF-1α promoter in TPH-1 cells, indicating a strong interaction between the two. This observation further underscores the crucial role of SETD2 in regulating HIF-1α expression through histone modification, specifically H3K36me3 (Fig. 2G). To explore further into the regulatory mechanism of SETD2 on HIF-1α expression, we utilized the HumanTFBD website (http://bioinfo.life.hust.edu.cn/) to retrieve the HIF-1α promoter sequence and identify potential H3K36me3 binding sites within the HIF-1α promoter region (Fig. 2H). The luciferase reporter assay outcomes revealed that in TPH-1 cells where H3K36me3 levels are depleted, the HIF-1α promoter-wt exhibits reduced relative luciferase activity compared to the HIF-1α promoter-mut (Fig. 2I). This observation reinforces the conclusion that H3K36me3 plays a pivotal role in the regulation of HIF-1α expression by binding to the HIF-1α promoter. The findings imply that SETD2 represses HIF-1α expression by promoting the H3K36me3 modification on the HIF-1α promoter.

### Silencing of HIF-1α abrogated the M1 macrophage polarization and glycolysis induced by SETD2 silencing in TPH-1 cells

**Figure 3:**
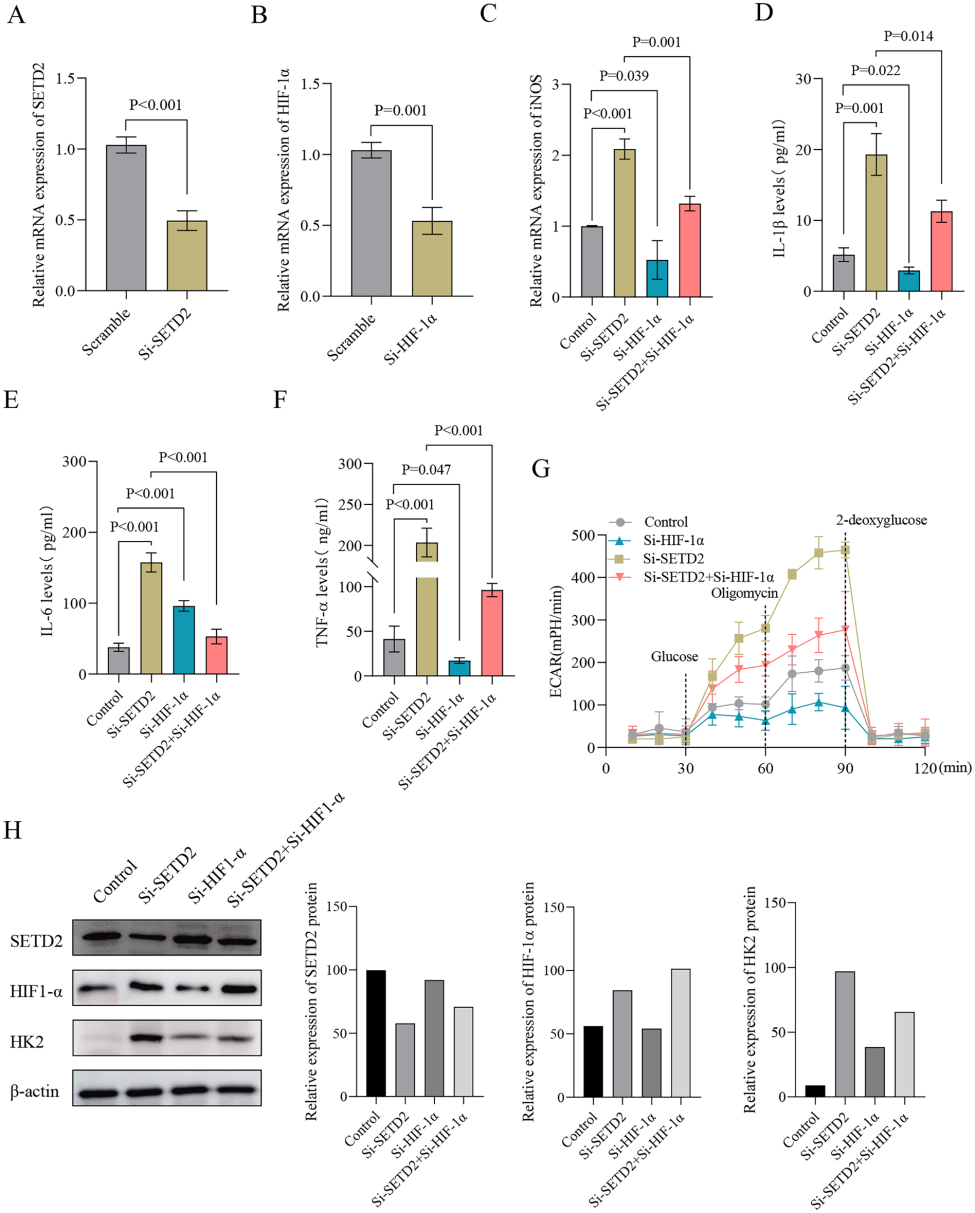
The silencing of HIF-1α abrogates the M1 macrophage polarization and glycolysis induced by SETD2 silencing in THP-1 cells. (**A**) THP-1 cells were genetically manipulated with a plasmid designed to specifically knockdown SETD2 expression, or a control plasmid with no silencing capacity. (**B**) The expression levels of HIF-1α were quantified by qPCR and further validated by western blot analysis. (**C**–**F**)At 48 h post-transfection, iNOS mRNA levels were measured by qPCR, while the levels of cytokines such as IL-1β, IL-6, and TNF-α in the cell supernatant were determined by ELISA. (**G**) At 48 h following transfection, ECAR levels were assessed using a Seahorse Bioscience extracellular flux analyzer. (**H**) Illustrative western blot images depicting the expression patterns of SETD2, HIF-1α, and HK2 at 48 h post-transfection are presented.

To gain further clarity on whether HIF-1α mediates the regulatory function of SETD2 on macrophage polarization and glycolysis in TPH-1 cells, the researchers transfected siRNA against SETD2 or HIF-1α into TPH-1 cells individually (Fig. 3A, B). After 48 h of transfection, the polarization of macrophages and glycolysis in TPH-1 cells were evaluated. The findings revealed that the silencing of SETD2 stimulated the expression of iNOS and enhanced the secretion of IL-1β, IL-6, and TNF-α in TPH-1 cells. Notably, the silencing of HIF-1α abrogated these effects induced by SETD2 silencing (Fig. 3C–F). Additionally, following SETD2 silencing, an increase in ECAR was observed, which was effectively counteracted by the silencing of HIF-1α (Fig. 3G). Furthermore, the knockdown of SETD2 led to elevated levels of HIF-1α and HK2; however, these elevations were abrogated by the silencing of HIF-1α. (Fig. 3H). Consequently, the suppression of HIF-1α attenuated the M1 macrophage polarization and glycolysis induced by SETD2 silencing in TPH-1 cells.

### SETD2 silencing in macrophage promotes hBMSCs osteogenic differentiation in co-cluture macrophage and hBMSCs in vitro

**Figure 4:**
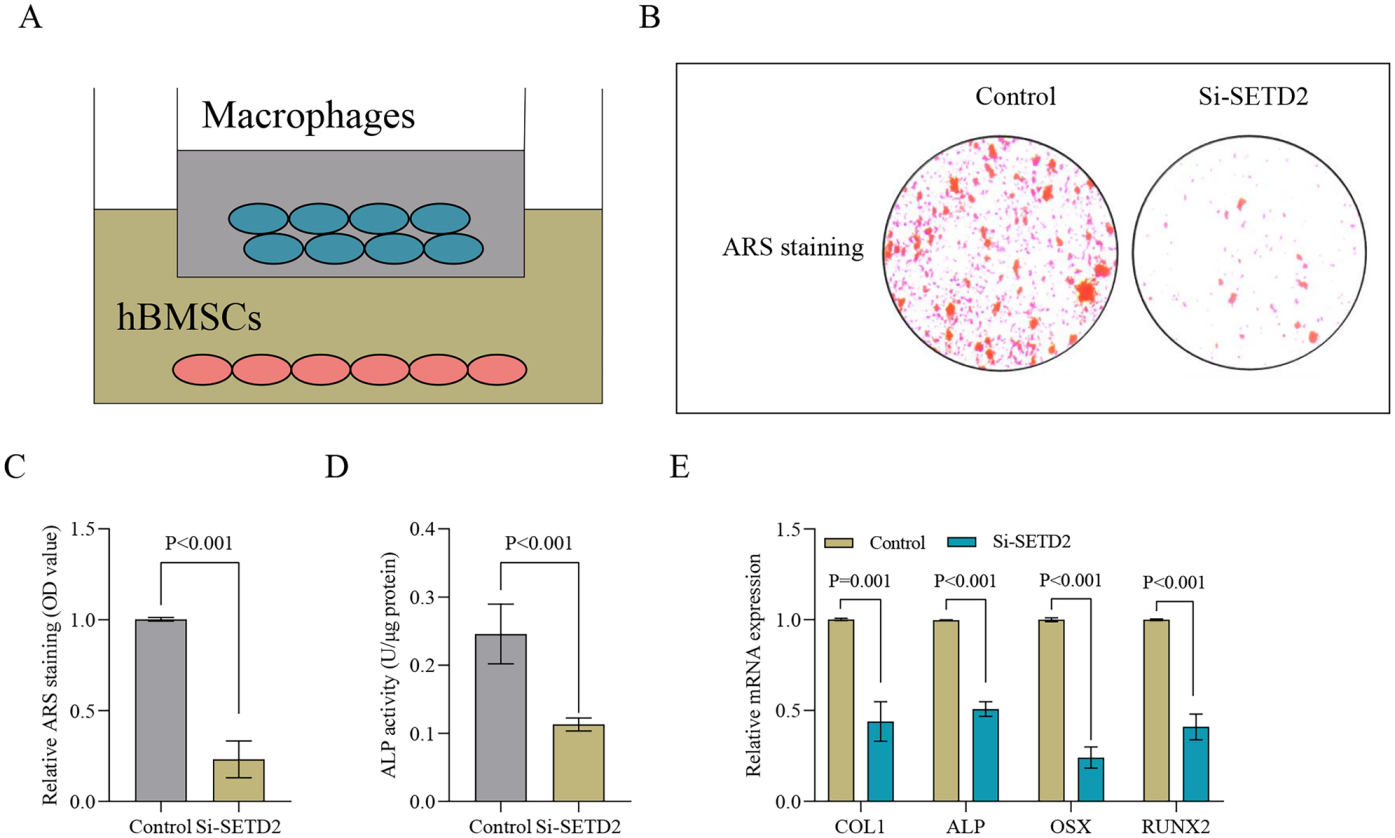
Downregulation of SETD2 in macrophages exerts an osteogenic oifferentiation effect on hBMSCs in a co-culture system. (**A**) The lower chamber was seeded with hBMSCs, whereas the upper chamber housed the macrophages for co-culture experiments. (**B**,**C**) To visualize calcium deposits in a co-culture system, ARS staining was conducted on cells transfected with SETD2 siRNA, as well as on non-transfected control cells. (**D**) ALP activity assays were conducted to assess osteogenic differentiation in a co-culture system transfected with SETD2 siRNA or not. (**E**) On day 7 of osteogenic differentiation of hBMSCs in a co-culture system, mRNA expression levels of COL1, ALP, OSX, and RUNX2 were quantified by qPCR in cells transfected with SETD2 siRNA compared to non-transfected controls.

The co-culture system employed allowed for the seeding of SETD2-silenced or non-silenced TPH-1 cells in the upper chamber and the cultivation of hBMSCs in the lower chamber (Fig. 4A). Upon SETD2 knockdown, a decrease in both ARS staining and ALP activity was observed, indicating altered osteogenic differentiation, compared to the control group after a 7 day co-culture period (Fig. 4B–D). Furthermore, statistical analysis of the mRNA expression levels of osteogenic marker genes, including COL1, ALP, OSX, and RUNX2, reinforced these observations (Fig. 4E). These results suggest that silencing SETD2 may hinder osteogenesis in hBMSCs when they are co-cultured with macrophages.

### SPA‑induced M1 macrophage polarization in co-culture macrophage and hBMSCs in vitro

**Figure 5:**
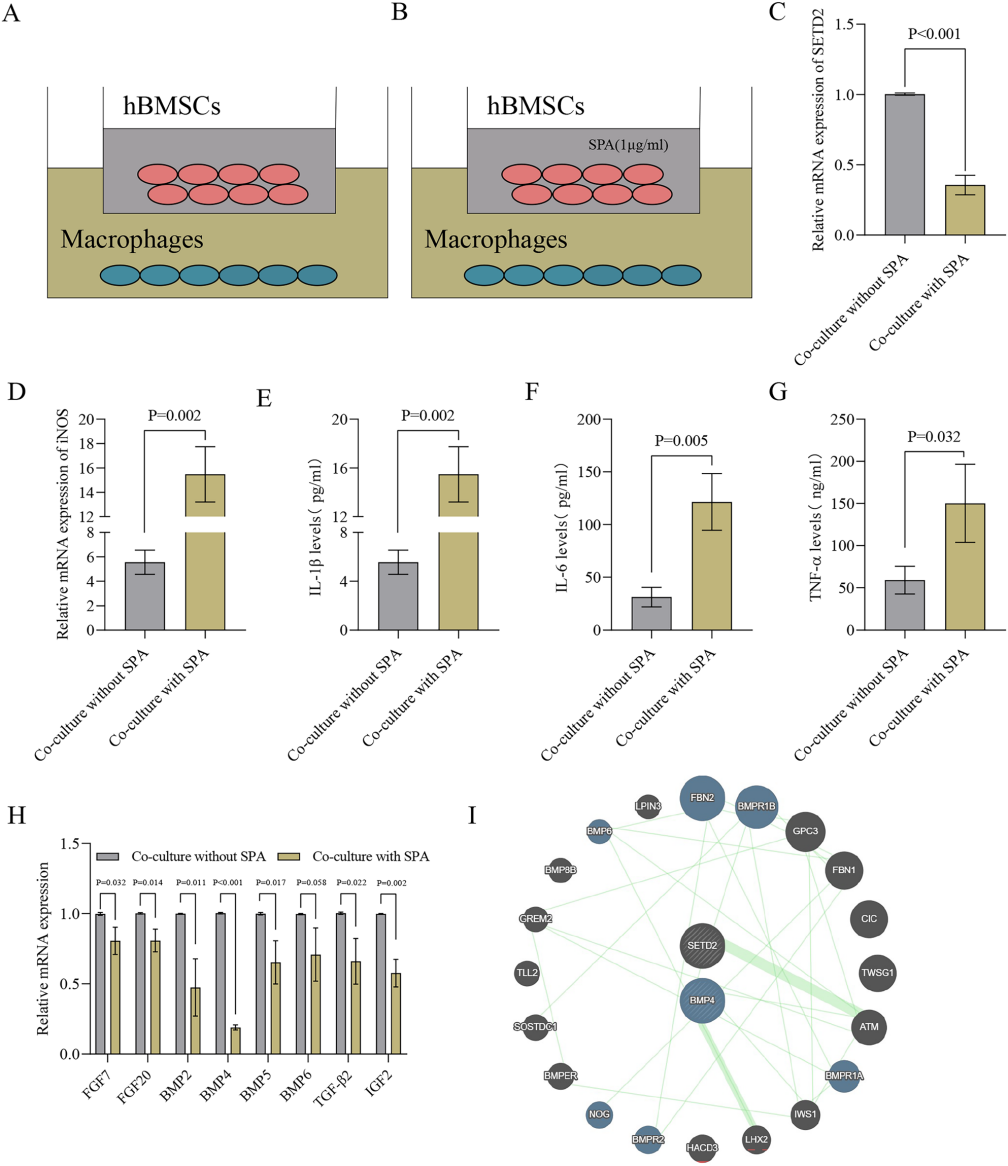
SPA-induced M1 macrophage polarization in co-Cultured macrophages and hBMSCs in vitro*.* (**A**) The macrophages were placed in the lower chamber, while the hBMSCs were positioned in the upper chamber, establishing an inverted co-culture configuration. (**B**) Macrophages were seeded in the lower chamber, and hBMSCs were cultured in the upper chamber with the addition of SPA to induce osteogenic differentiation. (**C**) qPCR was utilized to assess SETD2 mRNA levels across various groups. (**D**–**G**) qPCR was used to measure iNOS mRNA levels, while ELISA was employed to quantify IL-1β, IL-6, and TNF-α levels in the cell supernatant at 48 h post-transfection, comparing different experimental groups. (**H**) During co-culturing with hBMSCs, macrophages were identified as producers of osteogenic differentiation cytokines, including FGF7, FGF20, BMP2, BMP4, BMP5, BMP6, TGF-β2, and IGF2. (**I**) Co-expression of SETD2 and BMP4 at the protein level obtained from the STRING database.

Utilizing a co-culture setup, hBMSCs were seeded in the upper chamber, either alone or in combination with SPA, while TPH-1 cells were cultured in the lower chamber, allowing for interactions between the two cell types (Fig. 5A, B). It was observed that SETD2 expression decreased when co-cultured with SPA (Fig. 5C). Furthermore, iNOS, IL-1β, IL-6, and TNF-α were downregulated when macrophages and hBMSCs were co-cultured with SPA (Fig. 5D–G). It has been reported that cytokines promote osteogenic differentiation^18^. Hence, our objective was to delineate the cytokines that mediate the osteogenic differentiation of BMSCs through SETD2. We examined the expression of pro-osteogenic cytokines (FGF7, FGF20, BMP2, BMP4, BMP5, BMP6, TGF-β2, and IGF2) in macrophages following co-culturing with hBMSCs treated with SPA. Notably, BMP4 expression was significantly reduced (Fig. 5H). Using the STRING database, we found that SETD2 and BMP4 are co-expressed at the protein level (Fig. 5J). These findings suggest that SPA promotes M1 macrophage polarization by silencing SETD2, ultimately inhibiting osteogenesis in hBMSCs by regulating BMP4.

### SETD2 inhibitor EZM0414 rescues M1 macrophage polarization parameters in mouse model of osteomyelitis

Utilizing a murine osteomyelitis model induced by *Staphylococcus aureus*, we conducted a comprehensive investigation of the in vivo functions and mechanisms of HIF-1α and SETD2 in the development and progression of osteomyelitis. Following the initial *Staphylococcus aureus* vaccination, there was a notable loss of body weight on day 7 (Fig. S1A). Notably, SETD2 expression was reduced, while HIF-1α expression was elevated in bone marrow (Fig. S1B, C). Additionally, BMP4 levels were found to be decreased in bone marrow (Fig. S1D). On day 7, the infected group exhibited lower levels of SETD2 in bone marrow, along with heightened levels of HIF-1α, iNOS, IL-1β, IL-6, and TNF-α compared to the control group. These levels were only slightly increased by treatment with 50 mg/kg/day EZM0414 (Fig. S1E–J). The serum level of C-reactive protein (CRP) was also elevated by *Staphylococcus aureus* infection and dramatically decreased following EZM0414 treatment (Fig. S1K). Notably, BMP4 levels were downregulated in the mouse model of osteomyelitis when treated with EZM0414 (Fig. S1L). Finally, the mRNA expression levels of osteogenic marker genes, including COL1, ALP, OSX, and RUNX2, provided further confirmation of the aforementioned findings (Fig. S1M). Activation of macrophage M1 polarization in acute suppurative osteomyelitis of children.

**Figure 6:**
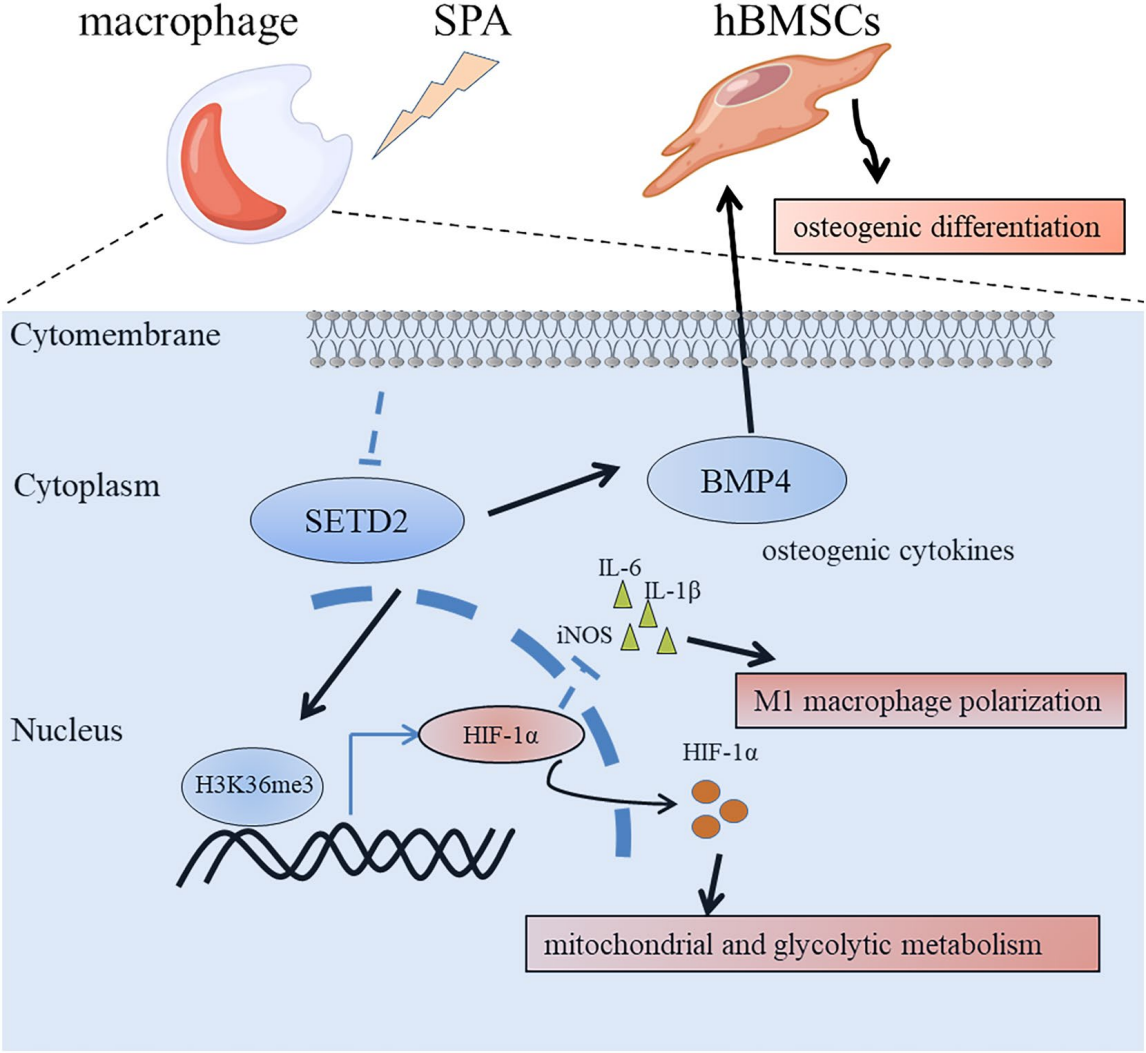
SPA inhibits hBMSC osteogenic differentiation and M1 macrophage polarization by suppressing SETD2 in acute suppurative osteomyelitis.

We collected clinical data from 12 children with acute suppurative osteomyelitis, and observed that the markers of M1 macrophage polarization, including A, B, and C, were all elevated (Fig. S2A–C). Additionally, CRP, a diagnostic marker reflecting bacterial infection, also showed an increase (Fig. S2D). These observations imply that HIF-1α and SETD2 play pivotal roles in the pathogenesis of osteomyelitis and could potentially serve as therapeutic targets for the treatment of this condition (Fig. 6).

## Discussion

Osteomyelitis, a debilitating bone disease caused primarily by *Staphylococcus aureus*, is particularly prevalent in the field of pediatric orthopedics^19^. This situation poses a grave danger to global public health, thus urgently requiring the development of therapeutic strategies to address osteomyelitis and its accompanying complications. Recent studies indicate that reprogramming the functional capabilities of macrophages could potentially serve as an effective therapeutic approach for osteomyelitis^6,20^.

To examine the role of SETD2 in macrophage function during osteomyelitis, we analyzed the expression levels of SETD2 in TPH-1 cells upon stimulation with SPA. We observed a decrease in SETD2 levels in TPH-1 cells treated with SPA. Furthermore, previous research conducted by Liu et al. demonstrated a downregulation of SETD2 expression in patients with inflammatory bowel disease^21^. In our study, we observed that the overexpression of SETD2 suppressed PSA-induced iNOS expression and reduced the production of cytokines such as IL-1β, IL-6, and TNF-α in macrophages. These proteins serve as markers of M1 macrophage polarization^22^. Based on our findings, we concluded that SETD2 has the ability to suppress M1 macrophage polarization induced by SPA. Our study is the first to report the suppressive effect of SETD2 on SPA-induced M1 macrophage polarization. As metabolic alterations in macrophages play a role in osteomyelitis^23^, we further investigated the impact of SETD2 on glycolysis in these cells. The findings revealed an elevation in glycolysis in macrophages, aligning with previous research^24^. Nevertheless, the overexpression of SETD2 suppressed glycolysis in macrophages. Previous studies have indicated that SETD2 may function as a novel regulator of the metabolic switch in clear cell renal cell carcinoma cells, promoting both mitochondrial and glycolytic metabolism^13^.

HIF-1α is known to regulate glycolysis in various diseases, including osteomyelitis^25^. Likewise, our findings demonstrated the activation of HIF-1α in TPH-1 cells upon stimulation with SPA. Consistent with our expectations, SETD2-mediated H3K36me3 modulation impacted HIF-1α expression, subsequently regulating M1 macrophage polarization and glycolysis. Although H3K36me3 is widely reported to facilitate transcription^26^. our study presents novel findings by demonstrating that SETD2-mediated H3K36me3 actually suppresses HIF-1α expression. However, the specific transcription factors involved remain to be determined. Prior reports have indicated that SETD2 depletion results in the downregulation of antioxidant genes, subsequently leading to an increased production of reactive oxygen species (ROS)^27^. These ROS play a crucial role in the regulation of both the M1 macrophage phenotype and HIF-1α. To date, our research is the pioneer in elucidating the regulatory role of SETD2 in governing M1 macrophage polarization and glycolysis, specifically through its modulation of HIF-1α. Furthermore, our study offers novel insights into the inhibitory mechanisms of SETD2 on osteogenic differentiation and M1 macrophage polarization in vivo.

Recent studies using co-culture systems and animal models have highlighted the significant role of macrophage and hBMSC cross-talk in bone regeneration^28^. Nevertheless, the precise mechanisms that underlie the communication between BMSCs and macrophages remain enigmatic. Multiple reports suggest that chemokines secreted by macrophages, along with osteoinductive factors, play a pivotal role in governing the recruitment and osteogenic differentiation potential of hBMSCs^29^. Our study demonstrated that macrophages play a significant role in promoting osteogenic differentiation of hBMSCs within a co-culture system. In a reciprocal manner, hBMSCs have been shown to modulate the polarization phenotype of macrophages^30^. Prior research has established that hBMSCs possess the capacity to suppress M1-like polarization while promoting M2-like polarization^30,31^. However, the precise mechanisms underlying how hBMSCs influence macrophage polarization remain enigmatic. In the current investigation, we observed a significant attenuation in the M1-like polarization phenotype in the absence of SETD2. Mechanistically speaking, when macrophages are co-cultured with hBMSCs, they regulate the expression of BMP4, thereby inhibiting osteogenic differentiation in the context of acute suppurative osteomyelitis. Hence, the modulation of M1-like macrophages via SETD2 could offer a promising novel therapeutic strategy for bone regeneration in acute suppurative osteomyelitis.

However, this study is not without its limitations. For instance, we did not conduct high-throughput sequencing, nor did we analyze the impact of SETD2 on osteoclast function or explore the effects of SPA on M2 macrophage polarization. To comprehensively understand the precise role of SETD2 in vivo, it is imperative to conduct further investigations using conditional knockout mice specifically targeting macrophages. Future research should also strive to identify the specific ligands associated with the paracrine effects of hBMSCs or the autocrine effects of macrophages. Given the intricate interplay between macrophages and hBMSCs, there are numerous other molecular components/pathways influenced by SETD2 that deserve further exploration. Additionally, there is a paucity of randomized trials comparing the relative benefits of different treatment modalities, highlighting the need for further clinical research in this area. In summary, our study has uncovered a previously unrecognized role of SETD2 in facilitating osteomyelitis recovery and augmenting osteogenic differentiation of hBMSCs within a co-culture system. This effect is linked to the activation of BMP4. Furthermore, this study reports for the first time that SETD2 suppresses M1 macrophage polarization and glycolysis by regulating HIF-1α via catalysis of H3K36me3 in the context of osteomyelitis.

### Supplementary Information

Supplementary Figure S1.

Supplementary Figure S2.

Supplementary Figures.

## Data Availability

The datasets utilized in this study are accessible upon reasonable request from the corresponding author.
