# Supplementary figures and images for: SPA inhibits hBMSC osteogenic differentiation and M1 macrophage polarization by suppressing SETD2 in acute suppurative osteomyelitis
Source: Sci Rep. 2024 Jun 3;14:12728. doi: 10.1038/s41598-024-63219-0 (PMC11148074; doi:10.1038/s41598-024-63219-0)

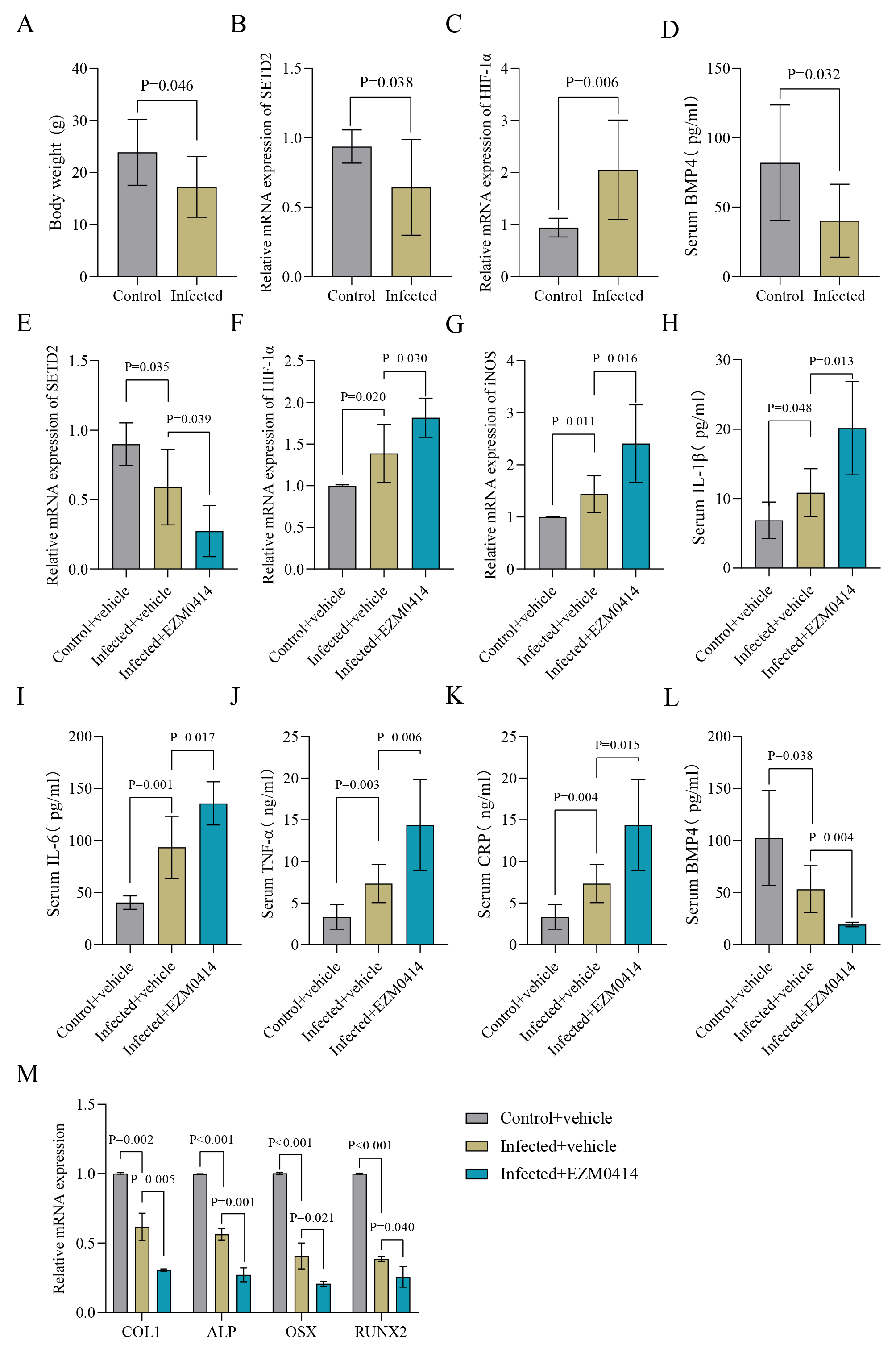

Supplement: Supplementary file 1 — Supplementary Figure S1. [file 41598_2024_63219_MOESM1_ESM.tif]

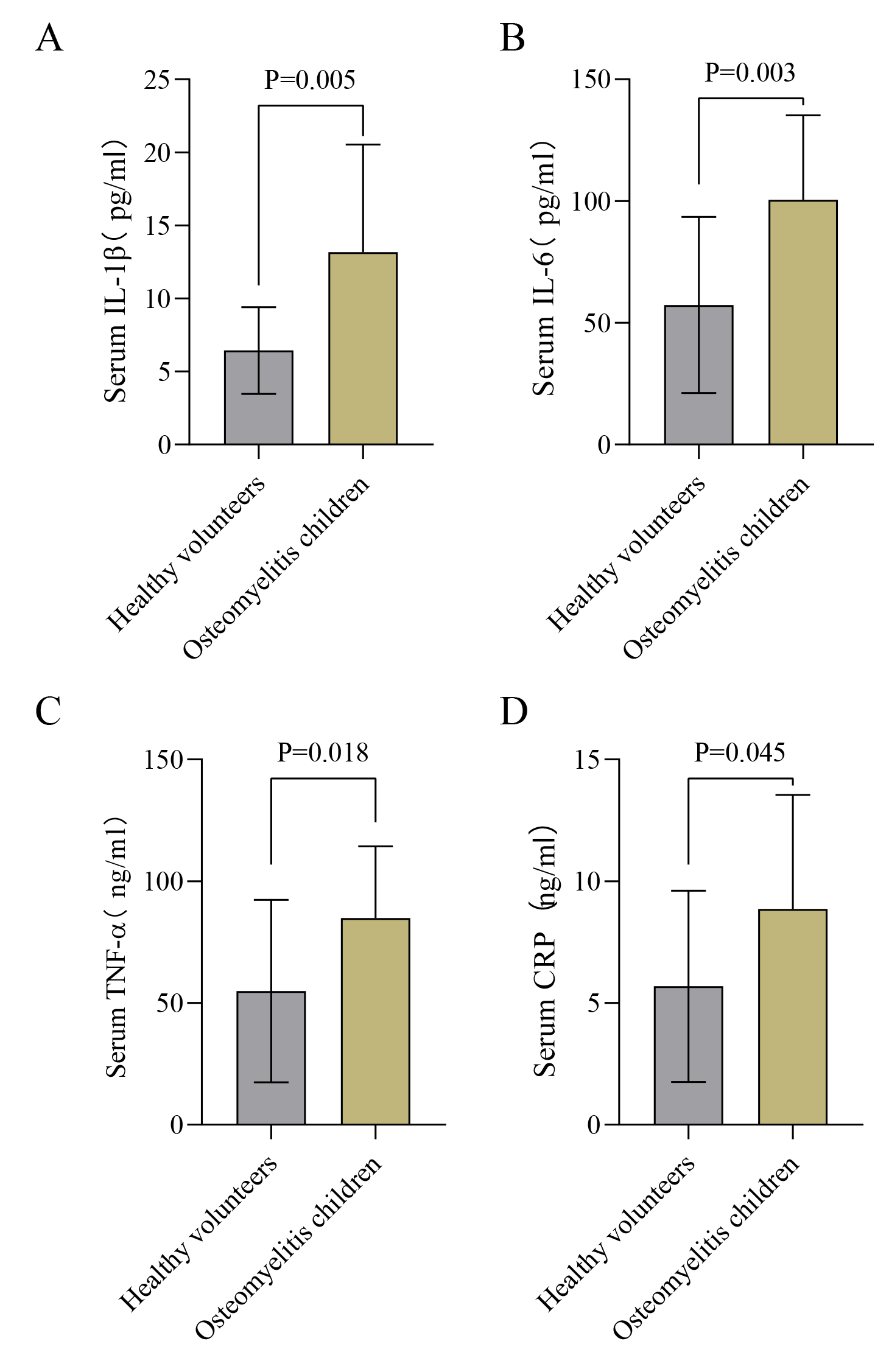

Supplement: Supplementary file 2 — Supplementary Figure S2. [file 41598_2024_63219_MOESM2_ESM.tif]
