# Supplementary material for: SPA inhibits hBMSC osteogenic differentiation and M1 macrophage polarization by suppressing SETD2 in acute suppurative osteomyelitis
Source: Sci Rep. 2024 Jun 3;14:12728. doi: 10.1038/s41598-024-63219-0 (PMC11148074; doi:10.1038/s41598-024-63219-0)

Fig 1c actin


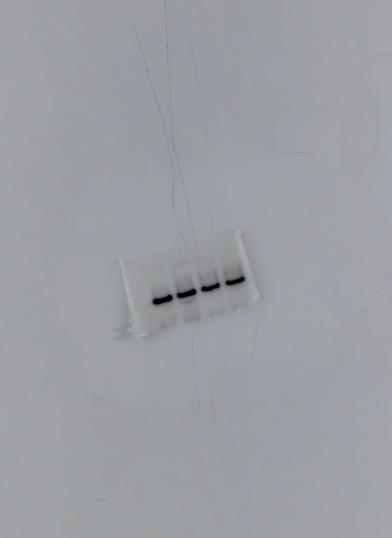

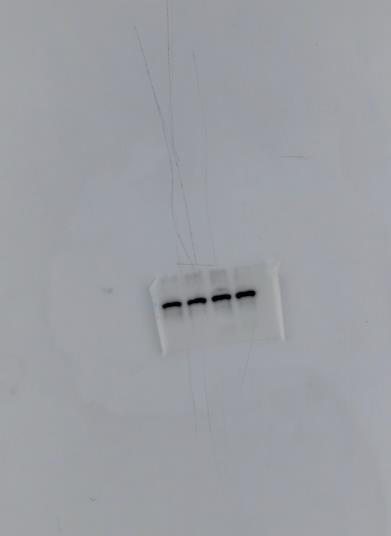

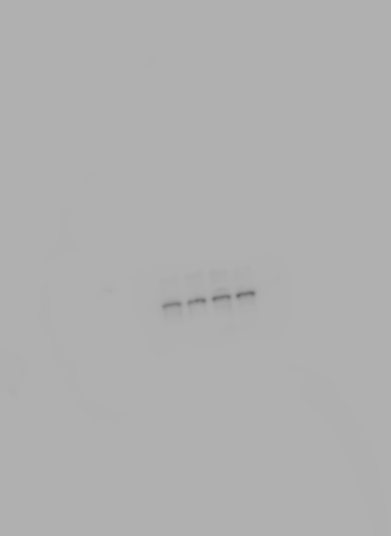


Fig 1c SETD2


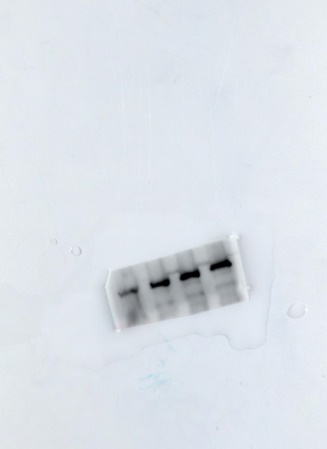

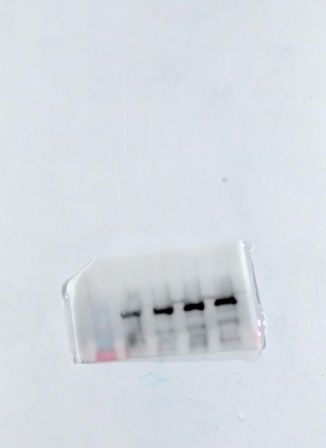

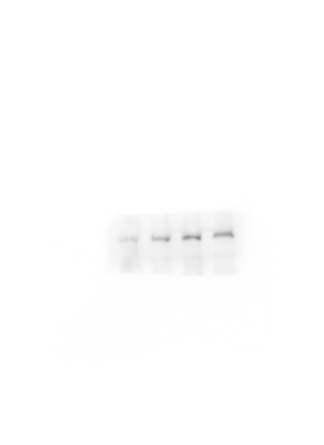


Fig 1j actin


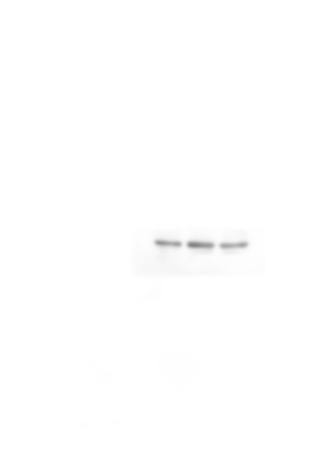

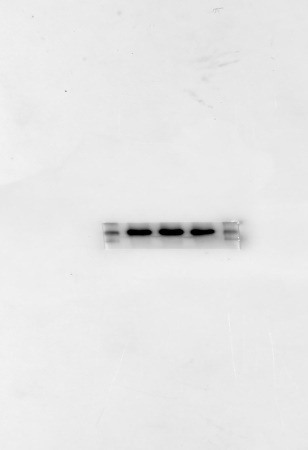

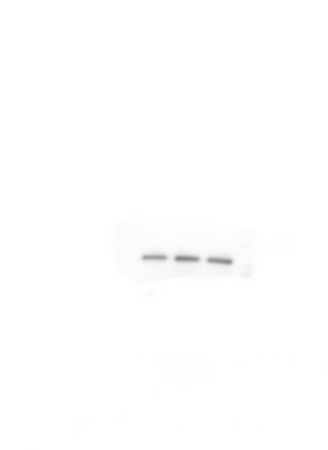


Fig 1j HIF


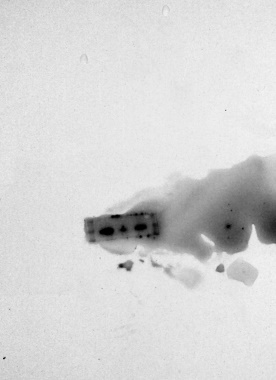

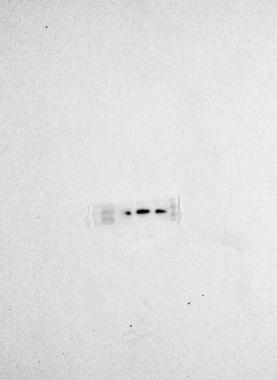

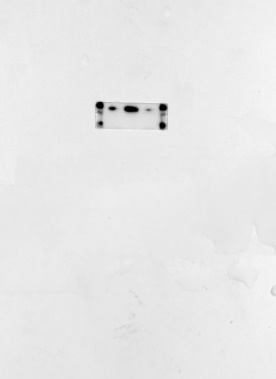


Fig1j HK2


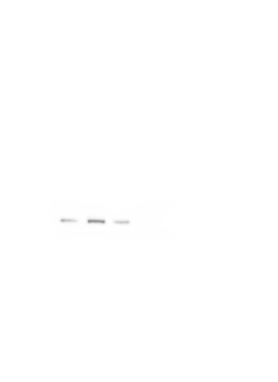

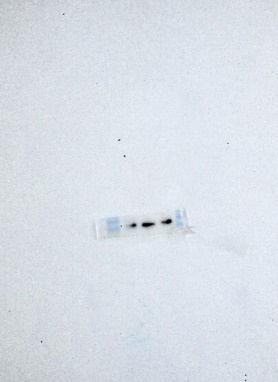

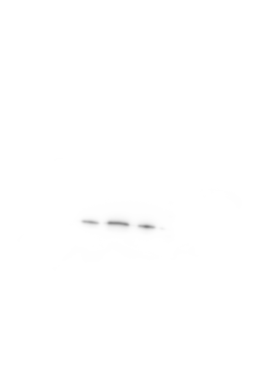


Fig1j SETD2


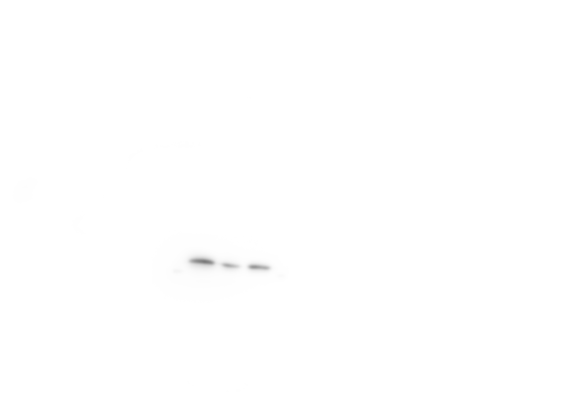

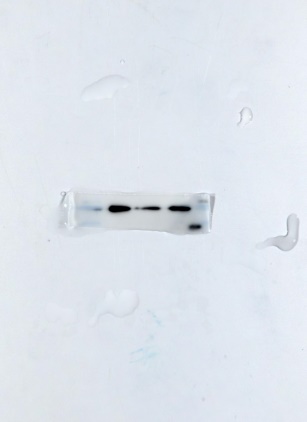

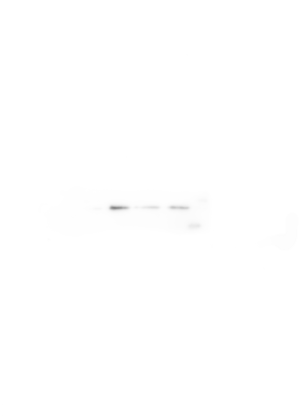


Fig2b actin


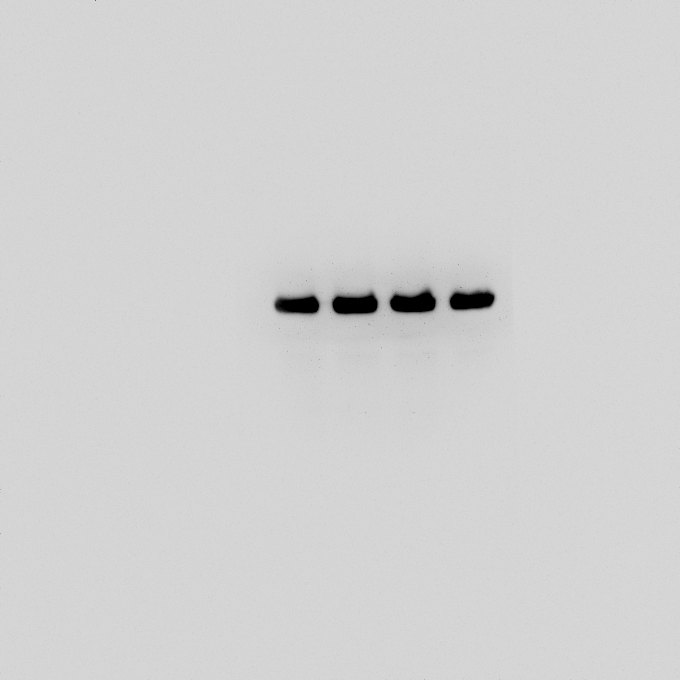

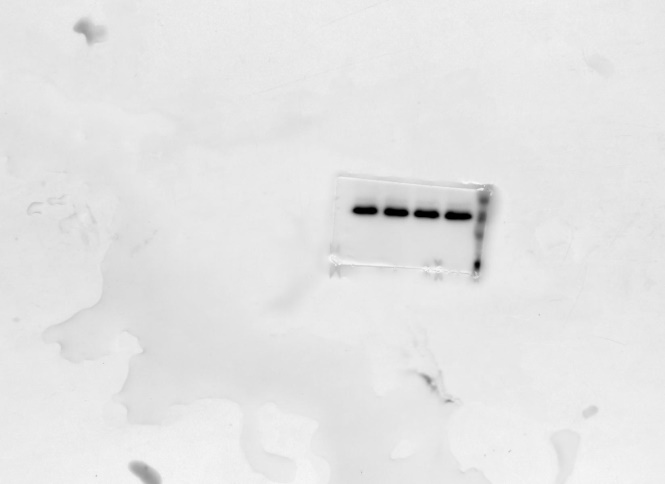

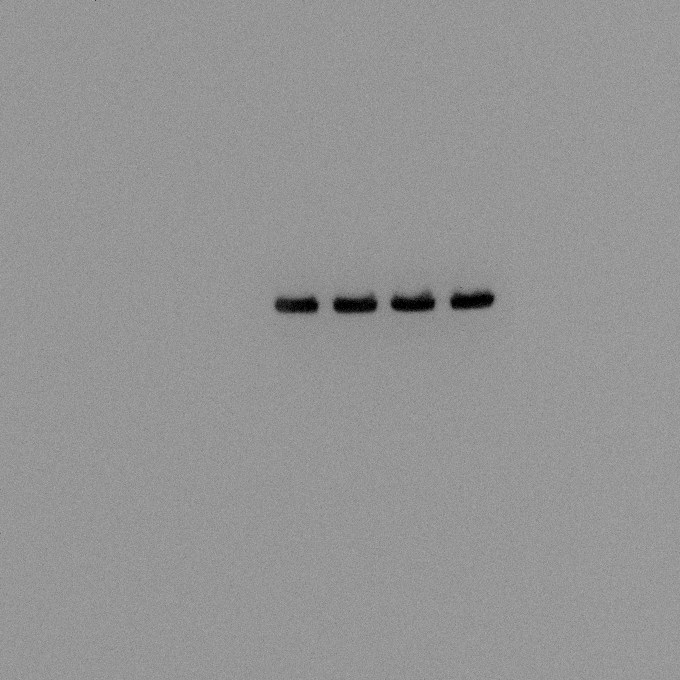


Fig2b HIF


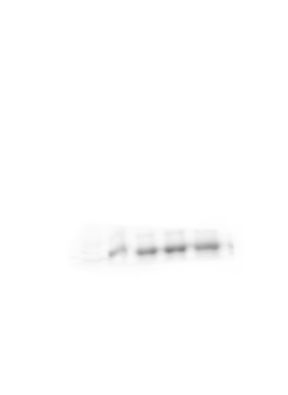

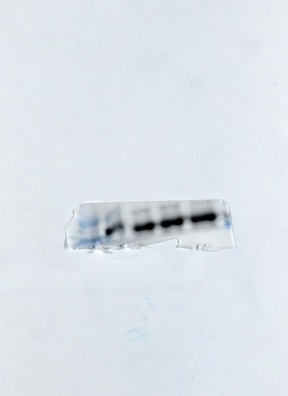

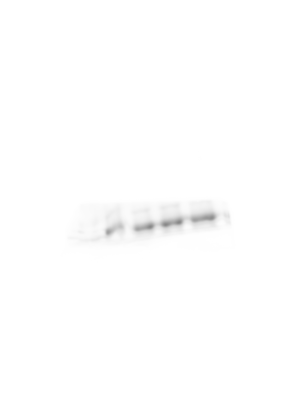


Fig2e H3


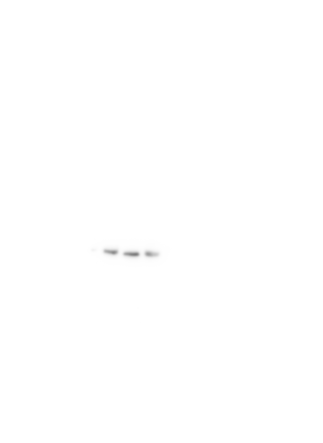

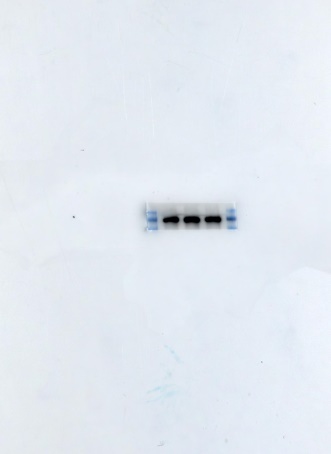

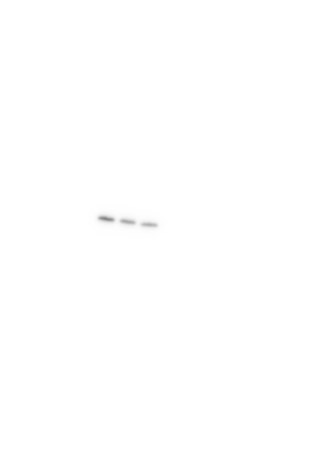


Fig2e H3K36


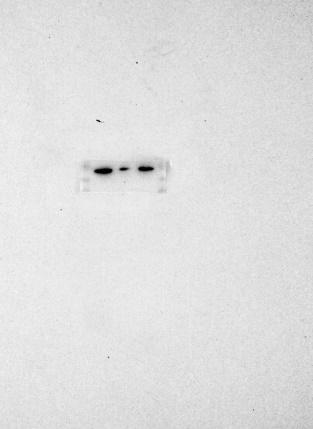

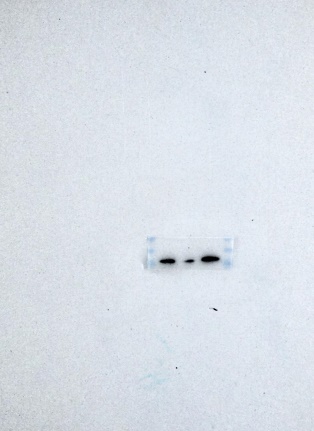

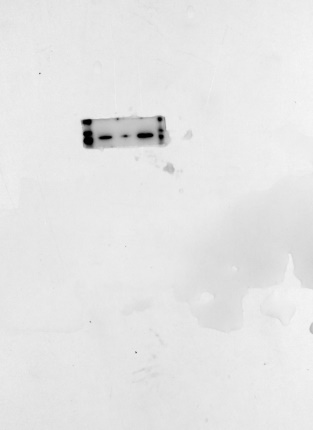


Fig3h actin


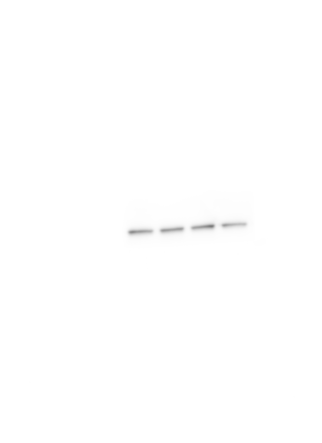

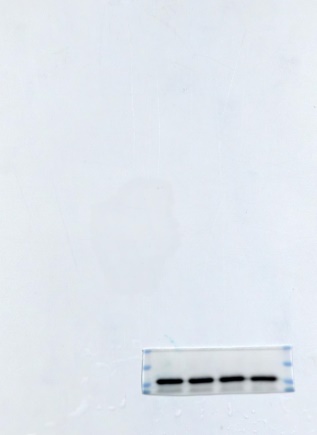

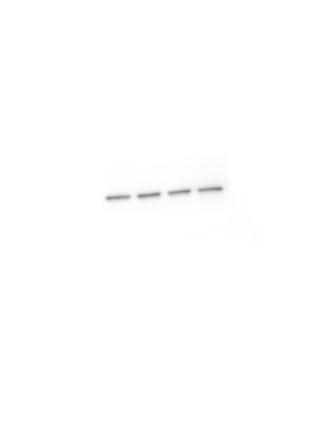


Fig3h HIF


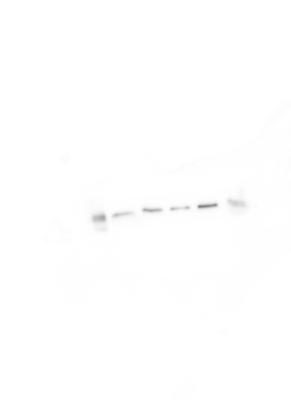

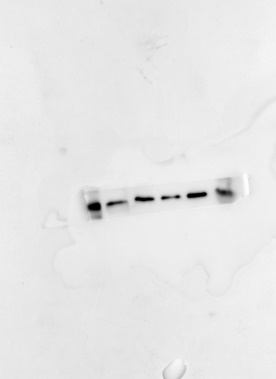

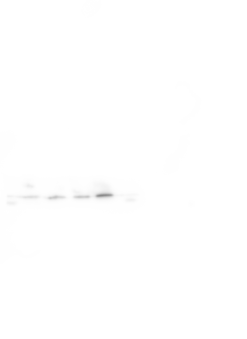


Fig3h HK2


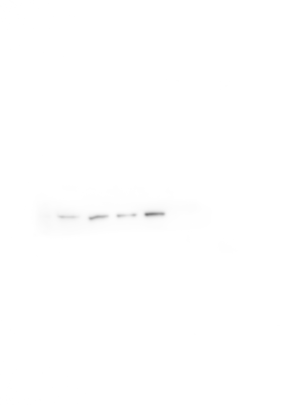

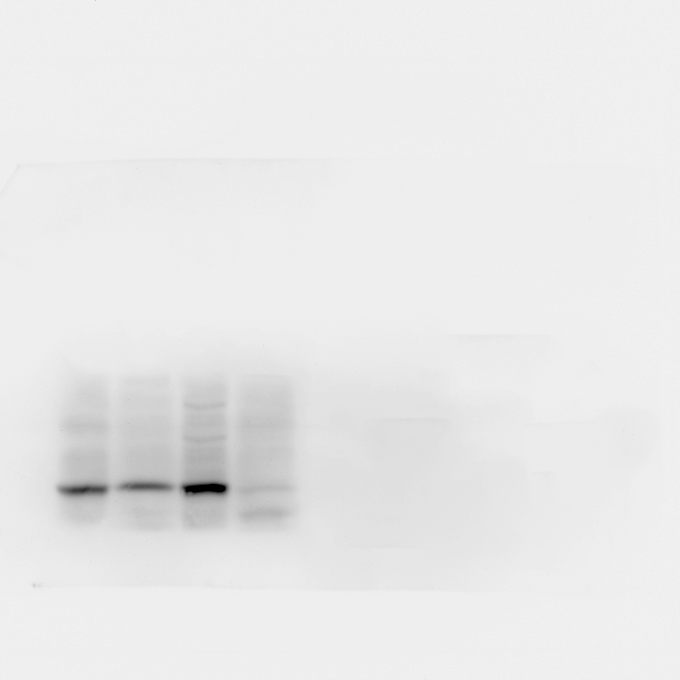

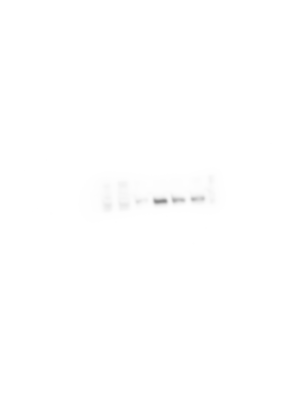


Fig3h SETD2


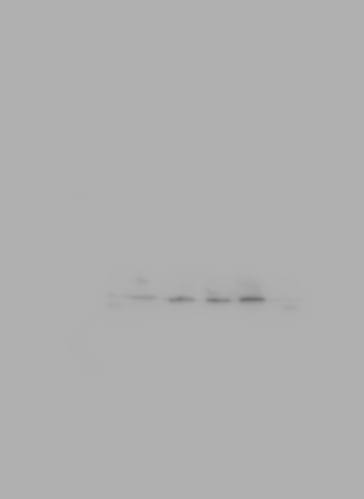

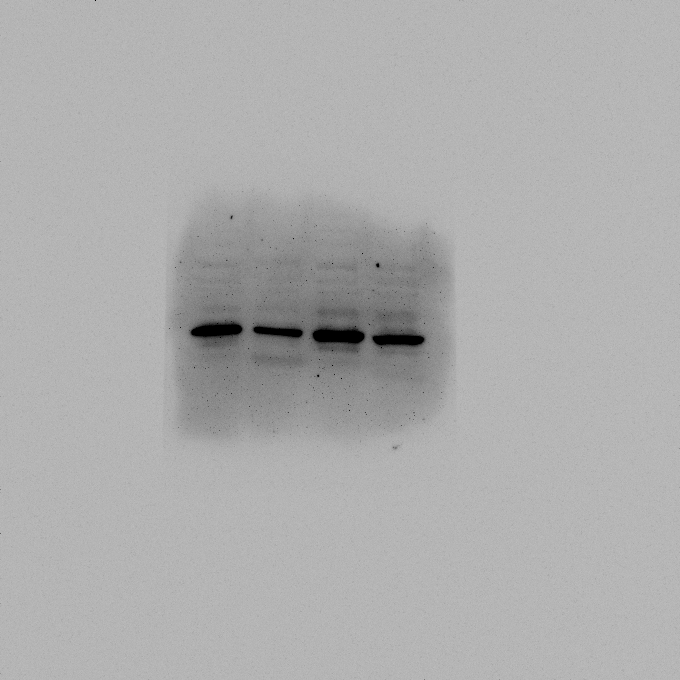

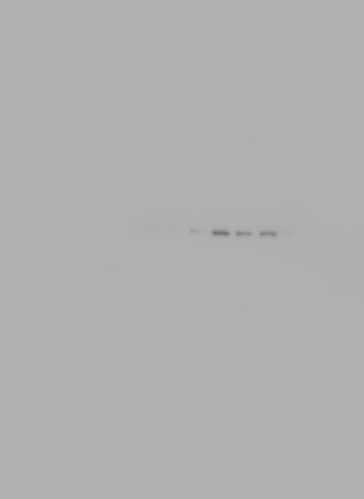

Supplement: Supplementary file 3 — Supplementary Figures. [file 41598_2024_63219_MOESM3_ESM.docx]
